# The Design and Engineering of a Fall and Near-Fall Detection Electronic Textile

**DOI:** 10.3390/ma16051920

**Published:** 2023-02-25

**Authors:** Zahra Rahemtulla, Alexander Turner, Carlos Oliveira, Jake Kaner, Tilak Dias, Theodore Hughes-Riley

**Affiliations:** 1 Nottingham School of Art & Design, Nottingham Trent University, Bonington Building, Dryden Street, Nottingham NG1 4GG, UK; 2 School of Computer Science, University of Nottingham, Jubilee Campus, Wollaton Road, Nottingham NG8 1BB, UK

**Keywords:** electronic textiles, E-textiles, electronic yarn, smart textiles, older people, fall detection, near-fall detection, machine learning, activities of daily living, design

## Abstract

Falls can be detrimental to the quality of life of older people, and therefore the ability to detect falls is beneficial, especially if the person is living alone and has injured themselves. In addition, detecting near falls (when a person is imbalanced or stumbles) has the potential to prevent a fall from occurring. This work focused on the design and engineering of a wearable electronic textile device to monitor falls and near-falls and used a machine learning algorithm to assist in the interpretation of the data. A key driver behind the study was to create a comfortable device that people would be willing to wear. A pair of over-socks incorporating a single motion sensing electronic yarn each were designed. The over-socks were used in a trial involving 13 participants. The participants performed three types of activities of daily living (ADLs), three types of falls onto a crash mat, and one type of near-fall. The trail data was visually analyzed for patterns, and a machine learning algorithm was used to classify the data. The developed over-socks combined with the use of a bidirectional long short-term memory (Bi-LSTM) network have been shown to be able to differentiate between three different ADLs and three different falls with an accuracy of 85.7%, ADLs and falls with an accuracy of 99.4%, and ADLs, falls, and stumbles (near-falls) with an accuracy of 94.2%. In addition, results showed that the motion sensing E-yarn only needs to be present in one over-sock.

## 1. Introduction

The purpose of this study was to design, engineer, and test a novel textile-based device for the monitoring of fall and near-fall events in older adults. Falls can be devastating to the quality of life of older people [1]. With a rise in the ageing population, falls will only continue to have a significant effect on older people, which will subsequently impact their health and social services. Injuries from falls are costly due to the treatment of the physical injury that can occur and its prolonged effects: after a hip fracture (a relatively common injury following a fall), only 36% of people are estimated to return to independent living [1]. Additionally, falls can cause a loss of confidence and a fear of falling, leading to frailty, which is a risk factor for falling [2], causing a continuous problem and reducing quality of life.

Consequently, being able to detect falls automatically has the advantage of reducing long falls, tracking how many times people are falling, and alerting medical professionals if required. A near-fall is defined as an event where a loss of balance (slip, trip, or stumble) occurs that can be corrected [3]. Studies have shown that near-fall can act as an independent indicator of risk of a fall [4]. Therefore, near-fall detection (detection of stumbling) would be of great benefit, as in the event of an increase in near-fall events, a care giver could be notified or medical professionals contacted, and a fall might be avoided.

Fall detection studies typically use wearable sensors, ambient (room based) sensors, or vision-based sensors for monitoring the movement of the participants [5,6,7]. Ambient and vision-based sensors work by detecting the environment around the user, typically indoors. The advantage of wearable sensors is that they can be used outdoors as well as indoors. Wearable sensors include inertial sensors (accelerometers, gyroscopes, and inertial motion units), smart watches, and smartphones. The most commonly used sensor for this type of study is an accelerometer [5]. There are fewer studies on near-fall detection [3], but these also commonly use accelerometers and inertial motion units (IMUs) to monitor the movement of the wearer. All of the studies within the literature on fall and near-fall have a focus on creating the most accurate machine learning algorithm for detection and have not focused on the sensing device.

Electronic textiles (E-textiles) are an obvious choice for integrating a fall detection system, as they are unobtrusive, comfortable, and hidden. These are desirable qualities for older people. Critically, comfort would be important to user compliance with wearing such a device for a prolonged period. To the knowledge of the authors, textile-based fall detection systems do not currently exist. This work focuses on creating an E-textile fall detection system for women, as they are often overlooked when the technology is gender neutral [8]. Additionally, the probability of non-fatal falls for women is higher, and they are more likely to fall than men [8], which can negatively impact their quality of life [8,9]. As such, for this study, the developed E-textile system was only tested on women.

This work used electronic yarn (E-yarn) technology to incorporate an inertial motion unit (IMU) into textiles. E-yarns technology allows any kind of small-scale electronic chip to be integrated within a textile structure without significantly impeding on its textile characteristics, and E-yarns for a variety of sensing applications have been presented in the past (including for motion detection [10,11]).

The research reported here is based on a pilot study to find the optimal placement of an inertial motion unit (IMU) for fall detection [12], which identified the most suitable sensor sites as being the thigh, ankle, and waist. This informed interviews and workshops held with older people, and human centered design was used to develop the E-textile prototype [13] (a full recounting of this research will be the subject of a future article). As such, E-yarns were used to engineer an over-sock.

The over-sock was used in a human trial as a proof-of-concept that a motion sensing E-yarn can be integrated into a garment and, in combination with a deep learning algorithm, detect falls. Each motion sensing E-yarn contained one commercially available IMU that measured acceleration in three axes and angular velocity in three dimensions. For the trial, 13 healthy women were recruited. Each participant performed three types of activities of daily living (ADLs), a near-fall (stumble), and three types of falls, whilst wearing an over-sock on each foot. The collected data from each foot was visually analyzed, and a machine learning algorithm was used to classify the data.

## 2. Materials and Methods

### 2.1. Hardware and Prototype

**Figure 1:**
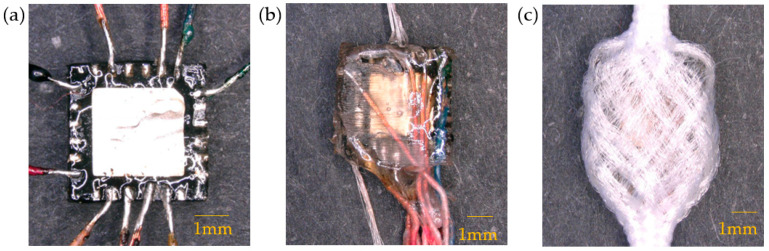
The IMU E-yarn. (**a**) The soldered stage of the E-yarn. (**b**) The second stage of the E-yarn, encapsulated with an added Vectran™. (**c**) The final stage of the E-yarn, showing the braided cover.

The motion sensing E-yarns created in this work used an embedded MPU-6050 IMU (InvenSense TDK, San Jose, CA, USA): This was a 6-axis IMU that included a 3-axis accelerometer and a 3-axis gyroscope. The MPU-6050 embedded E-yarns were manufactured using a three-step process shown in Figure 1. To begin with, ten insulated Litz wires (BXL2001, OSCO Ltd., Milton Keynes, UK) were manually soldered onto the IMU using a contact soldering iron. The soldered pads were informed by the MPU-6050 datasheet. The second step involved the encapsulation of the soldered IMU and a supporting Vectran™ yarn (Kuraray, Tokyo, Japan) within a UV curable resin (Dymax 9001-E-V3.5; Dymax Corporation, Torrington, CT, USA) micro-pod (4 mm × 4 mm × 1 mm). The Litz wires were all positioned in one direction, parallel to the Vectran™ yarn (see Figure 1b). The final step in the manufacturing process required the encapsulated IMU, along with the wires and Vectran™, to be covered in a braided sheath to consolidate the final yarn and add strength. This was achieved using a braiding machine (lay length = 5; RU1/24-80, Herzog GmbH, Oldenburg, Germany) and 24 polyester yarns (36 f/167 dtex; Ashworth and Sons, Cheshire, UK). This resulted in a final E-yarn that was 5.4 mm at its widest point, was mechanically robust, and had the appearance of a textile yarn (see Figure 1c).

The structure of the fiber braid was tight around the micro-pod, and previous work exploring vibration transmission through yarns indicated that such a structure would have a negligible effect on the transmission of vibration [10]; hence, it was not expected that the yarn structure would influence the recorded motion data, provided that tight textile structures were utilized. Regardless, the yarns were tested at each stage of the production process (after soldering, after encapsulation, after braiding) to ensure that they functioned in all dimensions at each stage (as breakages between the wires and solder pads could occur). Testing involved positioning the device relative to gravity in each axis to test the accelerometer and rotating the device at a known velocity (2 rad/s) using a custom apparatus in each axis to test the gyroscope. In all cases, the yarn production process had a negligible effect on the sensor’s performance; however, it should be noted that there were relatively large differences in readings from sensor to sensor (for example, the z-axis acceleration under gravity varied between 7.87–9.28 m/s^2^ depending on the E-yarn used). It was believed that this was due to the tolerances of the chip. In both the visual analysis and the deep learning analysis of the motion data, relative changes would be identified, so the differences in the absolute values recorded were not perceived as being an issue. It is important to highlight that looking at relative measurements removed certain operational considerations for using the over-sock, including hysteresis in the textile once the sock was put on (affecting the precise positioning of the sensor and potentially absolute measurements) and manufacturing tolerances.

The E-yarn was then integrated into the over-sock. The over-sock was seamlessly knit using three yarns (Stretchline 20/DCY Black N66 DD / 78/2 N6, Stretchline 16/SCY Black 60/60 N66, and Nylon6 70/68/2 Black) on a Stoll ADF 3 E7.2 knitting machine (Lengede, Germany). The sock included a knitted channel for the E-yarn to be inserted as well as a small, knitted pocket (30 × 60 mm) for the hardware unit (used to communicate with the embedded IMU and collect data). The hardware used for the human trials consisted of a microcontroller and a small operating circuit that was based on a recommended circuit design provided by the MPU-6050 datasheet to ensure the proper function of the IMU. The microcontroller used was an Itsy-Bitsy nRF52840 (Adafruit Industries, New York, NY, USA), and the operating circuit board was designed and produced at Nottingham Trent University. The operating board was connected to ten pins on the IMU (as informed by the datasheet), and four lines from this board were subsequently connected to the Itsy-Bitsy (3.3 V, ground, SCL, and SDA). The E-yarn had a connector soldered onto the end (20021311-00010T4LF, Amphenol Communications Solutions, Wallingford, Connecticut, USA) to allow for the removal of the supporting hardware for washing.

Data was collected using the Arduino 1.8.9 software (Somerville, MA, USA), at a 25 Hz sampling rate (informed by the pilot study and the literature [14]). The code for the microcontroller was developed using the Arduino Integrated Development Environment (Arduino, Turin, Italy). The Adafruit MPU6050 library (by Adafruit) was utilized in this work [15]. The data was read-in using the Arduino IDE and transferred to Microsoft Excel (Microsoft Corporation, Washington, DC, USA) using the ArduSpreadsheet plug-in (developed by Indrek Luuk, available here [16]), with Excel being used to create the graphs shown in Section 3.1 and Section 3.3. No filtering or signal processing of any kind was applied to the collected data.

Bluetooth was not used, as during the previously mentioned pilot study it was observed to be unreliable [12], and it was important to ensure the full collection of the data. It was believed that this may have been partially due to signal interference in the room used for the study. A microcontroller board with Bluetooth capabilities was selected to easily allow the innovation to communicate wirelessly in the future; however, developing this capability fully was beyond the scope of this pilot project.

**Figure 2:**
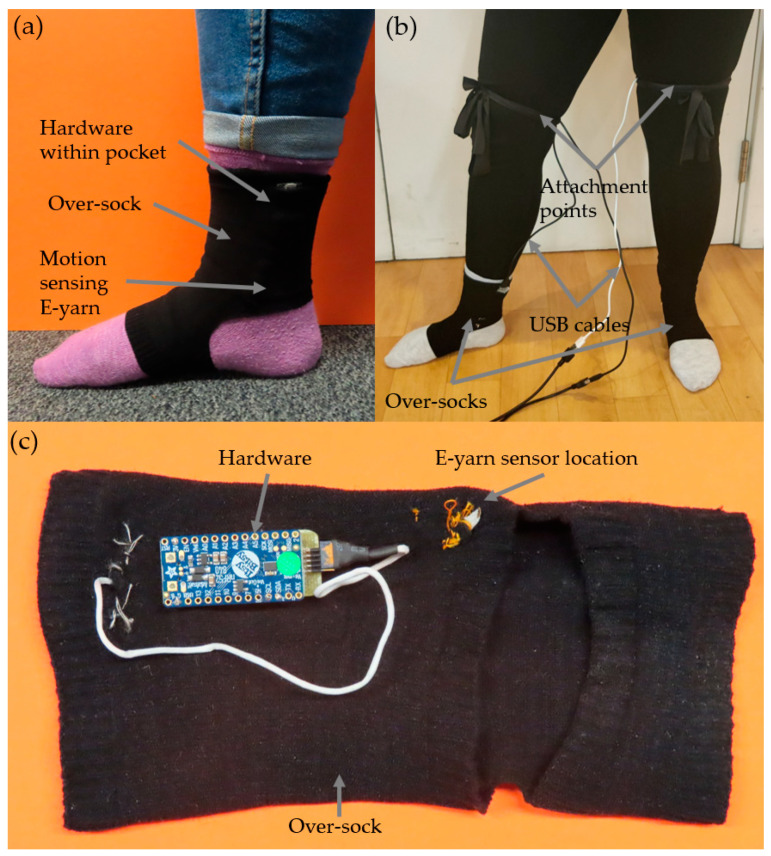
Photographs of the over-sock. (**a**) The over-sock contained one motion sensing E-yarn as well as the supporting hardware unit contained within a pocket. (**b**) Over-socks being worn during testing. The USB cables were attached to the participants using a stretchable fabric to prevent unnecessary strain on the hardware unit. (**c**) A photograph showing the inside of the over-sock. The hardware module has been removed from its pocket to better show its location within the sock. The E-yarn is secured within the socket with a few stiches (here shown using orange thread) to prevent movement.

Figure 2 shows images of the over-sock.

### 2.2. Testing Protocol

#### 2.2.1. Participants

For this study, thirteen healthy female volunteers, aged 22–33, were recruited. Their heights ranged from 1.57 m to 1.72 m and their weights ranged from 50 kg to 109 kg. Ethical approval was provided by the Nottingham Trent University Schools of Art and Design, Architecture, Design, and Humanities Research Ethics Committee. Informed consent was obtained by all the participants before the study.

#### 2.2.2. Activities

Each participant was asked to perform seven activities, repeated ten times: walking with a turn, sit-to-stand from a chair, “Timed Up and Go” a controlled stumble, and three types of falls onto a crash mat (sideways, backwards, and frontwards). Walking and the ability to stand from a seated position were chosen as they mimic basic ADLs [17]. The “Timed Up and Go” tests were included as they are used by clinicians for fall risk assessments [18]. The controlled stumble was included to simulate a near-fall. For the near-fall, each participant was given the freedom to choose how and when to stumble. They all interpreted the near-fall differently and performed the activity as they felt appropriate. Some walked, then stumbled and stopped for each repetition. Others walked in a straight line, stumbled, and continued walking in a line. Finally, some walked around the room, stumbled, and continued to walk around the room, with no pauses. The data from the controlled stumble therefore varied significantly from participant to participant as each participant interpreted a stumble slightly differently. The three types of falls were performed to see if there were significant differences in the results for different falls and to provide data to balance the datasets. For the fall activities, the participants stood directly next to the crash mat and fell onto it ten times. The data included each fall and each time the participants returned to a standing position. There was a pause before the participants fell, as they were lying down, and once they had returned to a standing position. The pause time varied between participants. The human trials were filmed so that movement could be easily identified in the data.

An over-sock was worn on both the left and right foot. Contained within each over-sock was a motion sensing E-yarn along with its own set of hardware that was connected to a laptop using extended USB cables (see Figure 2b).

### 2.3. Data Analysis and Machine Learning Algorthm

To classify the data gathered in this work, a deep learning classifier was used. The types of deep learning architecture that would be most applicable to the data in this work were those that were developed to classify time series data. One of the deep learning architectures that has been particularly adept at classifying time series data relating to movement and gait are variants of recurrent neural networks, more specifically BiLSTM’s (Bi-directional long term short term memory network) [11,19,20]. Due to its previous success in classifying time series data, the same BiLSTM was used in this work. The architecture of the network that was used to classify the data can be seen in Table 1.

The raw data collected from the sensors was fed directly into the network. The data was then split into data instances consisting of 200 timesteps (a timestep is an instance of data gathered at a specific time), which were sampled at intervals of 20 timesteps. The data was not down-sampled at this stage; collectively, the data instances covered all of the timesteps (i.e., the first data instance represented timesteps 1–200, the second 21–220, and so on). This aimed to ensure that a large volume of the features were present within the data, without generating excessive volumes of data. In total, this generated 17,138 data instances over all of the ADLs. This was randomly down sampled for all experiments to achieve a class balance between the ADL’s, except for the work in Figure 5, where it was important to understand how all of the ADL’s were classified. The data was split approximately into 75% training, 12.5% validation, and 12.5% testing data (which is the data available in the confusion matrices). The testing/validation data used was from three participants in the cohort, and the remaining data was used for training. This was done to best ensure the real-world applicability of this work by testing the network on participants that were not involved in the training data. The networks were trained for a maximum of 50 epochs or until the performance of the validation data had not improved for 10 subsequent epochs. The ADAM optimizer was used with a minibatch size of 128.

Results from the trained model are presented as confusion matrices: A confusion matrix is a visual representation of the performance of the algorithm. The column on the far right of the plot shows the percentages of all the examples predicted to belong to each class that are correctly and incorrectly classified. These metrics are often called precision (or positive predictive value) and false discovery rate, respectively. The row at the bottom of the plot shows the percentages of all the examples belonging to each class that are correctly and incorrectly classified. These metrics are often called the recall (or true positive rate) and false negative rate, respectively. The cell in the bottom right of the plot shows the overall accuracy. The values in the center of the matrix (red and green boxes) relate to classification with respect to the complete dataset and are used to determine the precision and recall.

## 3. Results

### 3.1. Visual Representation of the Data

**Figure 3:**
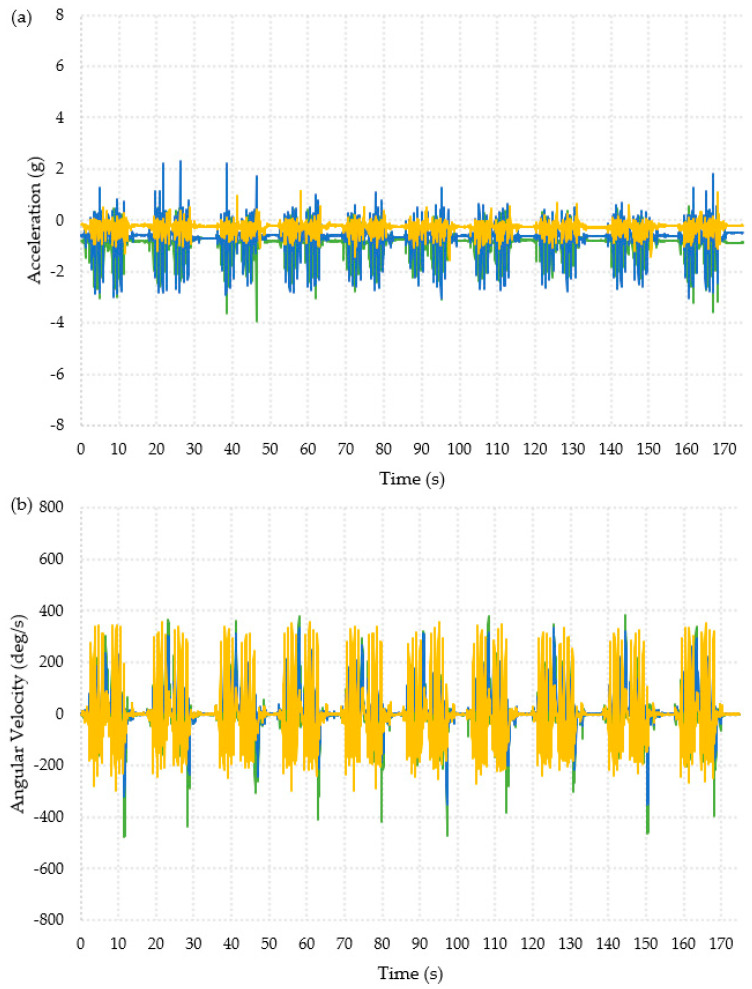
Graphical representation of the data collected from one foot for one participant during the Timed Up and Go activity, ten repeats of the Timed Up and Go are shown. X-axis = 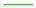, y-axis = 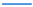, z-axis = 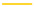. (**a**) Acceleration. (**b**) Angular velocity.

**Figure 4:**
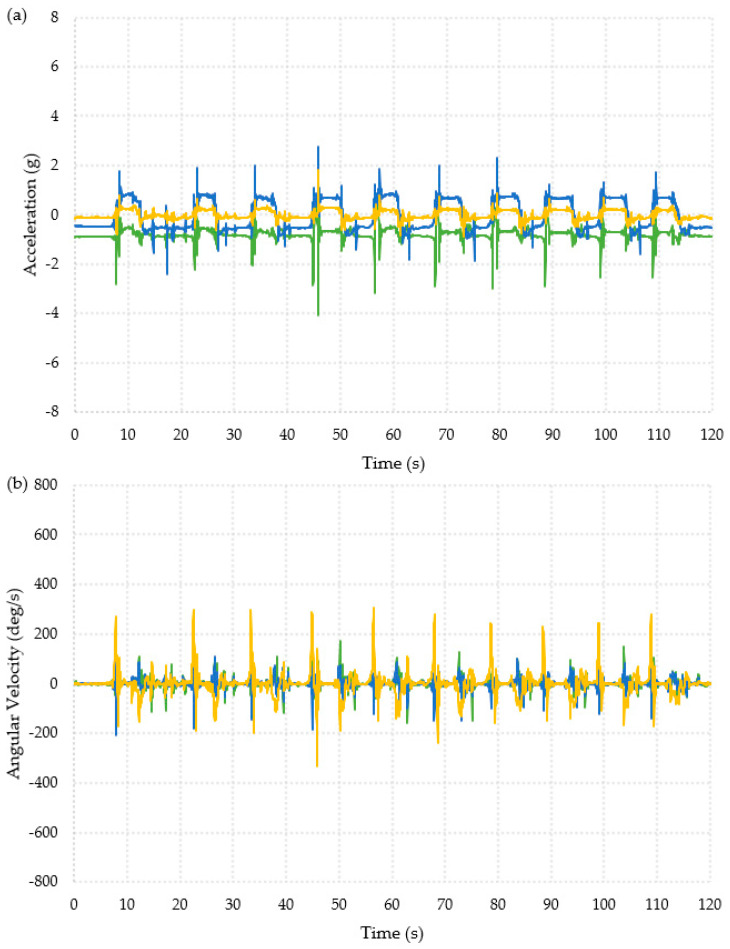
Graphical representation of the data collected from one foot for one participant during the backwards fall activity. X-axis = 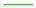, y-axis = 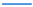, z-axis = 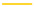. Ten repeated falls are shown. (**a**) Acceleration. (**b**) Angular velocity.

The collected data was first interpreted visually, as shown in Figure 3 and Figure 4. The data appeared to show similar patterns for each participant and for both feet (full datasets are available in the publicly available data-archive associated with this paper). The ADL presented in Figure 3 shows data from the “Timed Up and Go” test, which is a combination of the activities in the other two ADLs, and the data collected during a backwards fall are shown in Figure 4. The data used for these graphs was for the right foot of Participant 1.

In Figure 3a, the acceleration data was visible when the participant was moving compared to when they were still. The turns were also visible in Figure 3b, where there were two per repeat activity; this can be seen by looking at changes in the x- and y-axes. The second turn before the participant sat back down had the largest change in angular velocity, about −430 deg/s in the x-axis and 350 deg/s in the y-axis. The turns are masked within the acceleration data due to the walking motion. The sit-to-stand activity cannot be seen visually in the data.

The ten falls can clearly be seen from the acceleration data shown in Figure 4a. There was a change in acceleration on all three axes. From the first fall, there was an initial decrease in the x-axis of 1.95 g followed by an increase of 1.2 g. The y- and z-axes showed an increase in acceleration of around 1.3 g and 0.5 g, respectively. Figure 4b showed an increase in the angular velocity in the z-axis, with a maximum increase of about 299 deg/s at the point of the fall. There was also a decrease in the x- and y-axis angular velocities at the point of the fall. The x-axis decreases varied between 40 and 80 deg/s, and the y-axis decreases varied between 100 and 200 deg/s.

The patterns in Figure 3 and Figure 4 clearly showed very different behavior for each activity and might allow for thresholds to be defined to identify falls.

### 3.2. Using a Machine Learning Algorithm to Identify Falls

The collected data was used to train a deep learning model in the interest of accurately classifying the different activities. Full datasets were used in the model, and therefore the data included the pauses between each repetition of the ADLs and the participant getting back up after each fall. The stumble data did not have a strict pattern, as each participant did this activity differently. Some stumbled then paused, others walked in a line stumbled and turned, whilst some walked around the room and stumbled as they moved.

**Figure 5:**
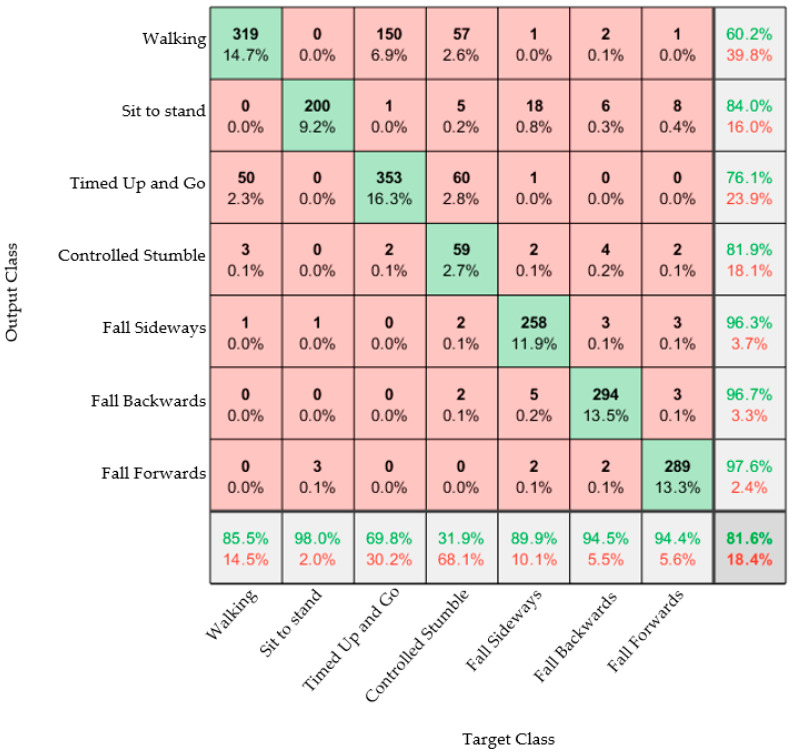
Confusion matrix classifying all of the activities performed for the data collected from all of the participants.

Figure 5 shows the confusion matrix generated when acceleration and angular velocity data from both feet was used. This data represents all 13 participants.

From Figure 5, an overall classification accuracy of 81.6% was observed. This also showed that the algorithm can easily classify between ADLs and falls. It was seen that the controlled stumble activity was the hardest to classify, which was to be expected. The data used to create the confusion matrix included all of the data for all of the participants. However, the fall acceleration data for Participant 10 was incomplete, and the sensor appeared to have broken during this trial. As the participant did not wish to repeat the fall experiments, this could not be repeated. Therefore, the other confusion matrices present below were created without Participants 10’s data and without the stumble. The confusion matrices below were trained using data from nine participants and tested on three participants.

**Figure 6:**
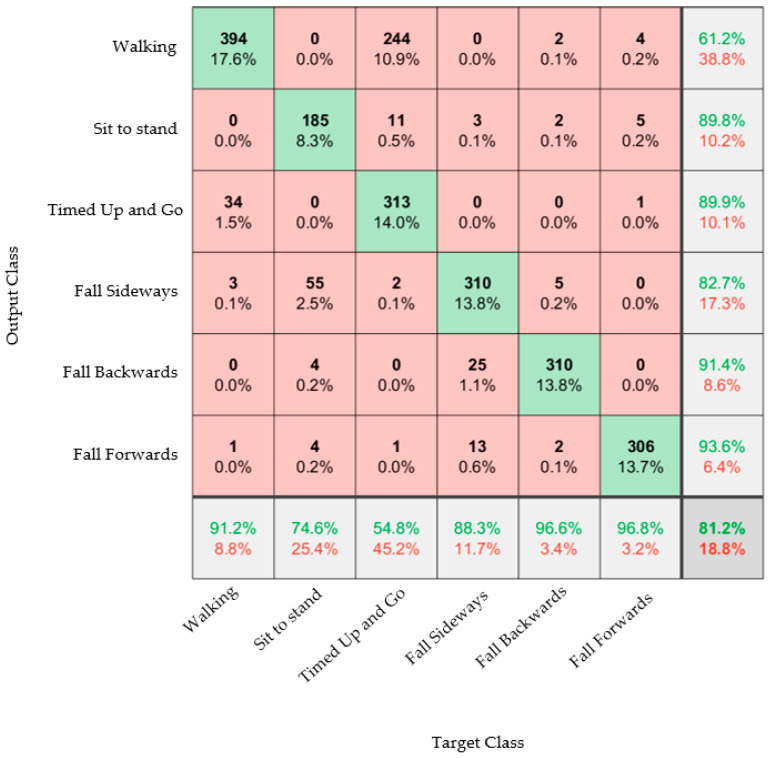
Confusion matrix showing the results of the classification of the three ADL activities and three fall activities.

Figure 6 shows that excluding Participant 10 and the stumble activity did not make a significant change to the overall accuracy of the model. An accuracy of 81.6% was seen in Figure 5 and 81.2% in Figure 6. The sideways fall could be classified with an accuracy of 88.3%, whereas the other two falls could be classified with accuracies of 96.6% and 96.8%; the reason for this discrepancy is unclear. It was also observed that there were some misclassifications between the Timed Up and Go tests and walking activities; this was likely due to the similarities between the activities. Ultimately, the point of the system was to distinguish between ADLs and falls, so this confusion would not be a hindrance in implementing the device.

**Figure 7:**
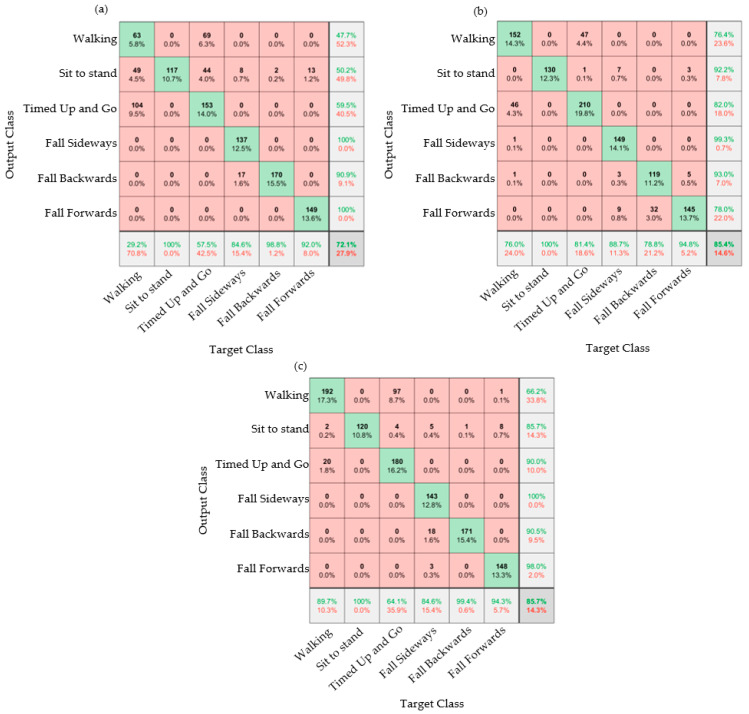
Confusion matrices for the data taken from the right foot. (**a**) Acceleration data. (**b**) Angular velocity data. (**c**) Both acceleration and angular velocity data.

It was subsequently desirable to further understand what data was helpful in classifying falls and ADLs. Nine conditions were explored by looking at the processing of data from both feet individually (as well as together) and by looking at all of the sensor data, acceleration data, and angular velocity data individually. Figure 7 shows the confusion matrices for the right foot only.

Figure 7 shows that using the angular velocity data on its own allowed for a more accurate classification when compared to using the acceleration data alone. The sit to stand for all three conditions (as shown in Figure 7a–c) was 100% accurate. As in Figure 6, the walking and timed up and go could sometimes be misclassified. This was because these activities are similar, and the majority of the timed up and go test consisted of walking and turning, which was also the case for the walking activity. In Figure 7a, it is seen that the acceleration data misidentifies walking as the timed up and go activity in a significant number of cases, with walking being correctly identified in only 47.7% of cases. However, as previously mentioned, it only mattered that the algorithm could identify falls and non-fall activities (ADLs). The classification of falls accurately (particularly the forward fall) was observed to be best when using either the acceleration data or a combination of the acceleration data with angular velocity data.

The table below compares the nine different iterations of the confusion matrices, exploring using data from the left, right, or both feet against combined (accelerometer and angular velocity) or individual measurements from the motion sensing E-yarn. These percentages are taken from confusion matrices in Figure 6 and Figure 7, with five additional matrices presented in the supplementary data (available in the data-archive).

The table showed that, generally, models trained using the angular velocity data were more accurate in classifying activities than those trained using the acceleration data. For the left foot, the confusion matrices showed that the acceleration data was more accurate for sideways falls; this was because all the participants fell on their right side. Therefore, the right foot did not move as much as the left (as observed in the videos of the activities). The use of the angular velocity data was generally better for the identification of falls. The left foot’s angular velocity data provided the most accurate classifications (87.9%), followed by the right foot’s combined acceleration and angular velocity data (85.7%). A key finding was that using data from one foot to train the algorithm often provided a more accurate classification than when data from both feet were used. It should be noted that the confusion matrices used to create Table 2 all showed the misidentification between the walking and timed up and go activities observed in Figure 4, Figure 5 and Figure 6.

**Figure 8:**
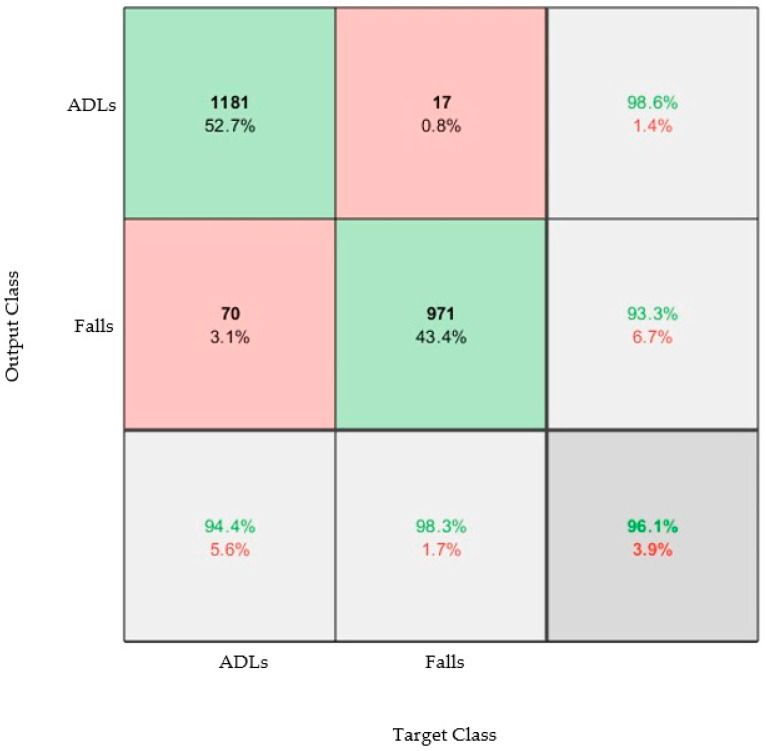
Confusion matrix that classifies between the three types of ADL (walking, sit to stand, timed up and go) and the three types of fall. The model was trained and tested using datasets from both feet and both the acceleration and angular velocity data.

While different activities could be identified with a high accuracy, for this device the identification of specific activities was not necessary, and instead only when the wearer was conducting an ADL or had fallen was really of interest. Figure 8 shows the confusion matrix that classifies between the ADLs (non-falls) and falls. The results showed a high accuracy of 96.1%. This result proved that the over-sock could be used to accurately detect falls.

**Figure 9:**
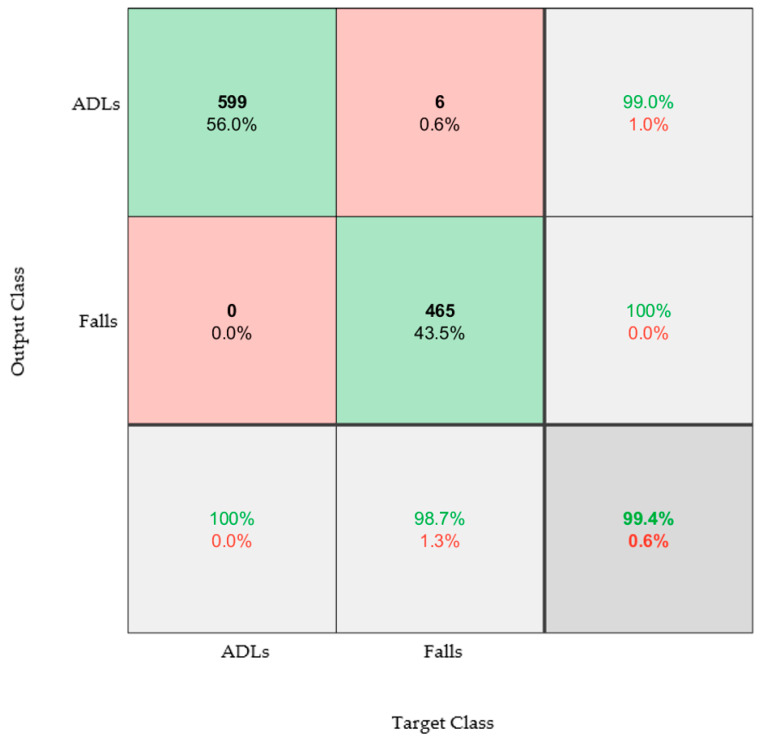
Confusion matrix that classifies between the three types of ADL (walking, sit to stand, timed up and go) and the three types of falls. The model was trained and tested using datasets from only the right foot and both acceleration and angular velocity data.

Previously, it was observed that taking measurements from only one foot improved the accuracy of the model. By taking data from only the right foot (informed by Figure 7 and Table 2), the model was re-trained, and the following confusion matrix was achieved (Figure 9).

An accuracy of 99.4% was achieved. Not only does this further evidence the suitability of the over-sock and algorithm for detecting falls, but it also showed that only one over-sock was sufficient. This is important for the implementation of such a system, as one over-sock is significantly less costly than two.

### 3.3. Controlled Stumble Data

**Figure 10:**
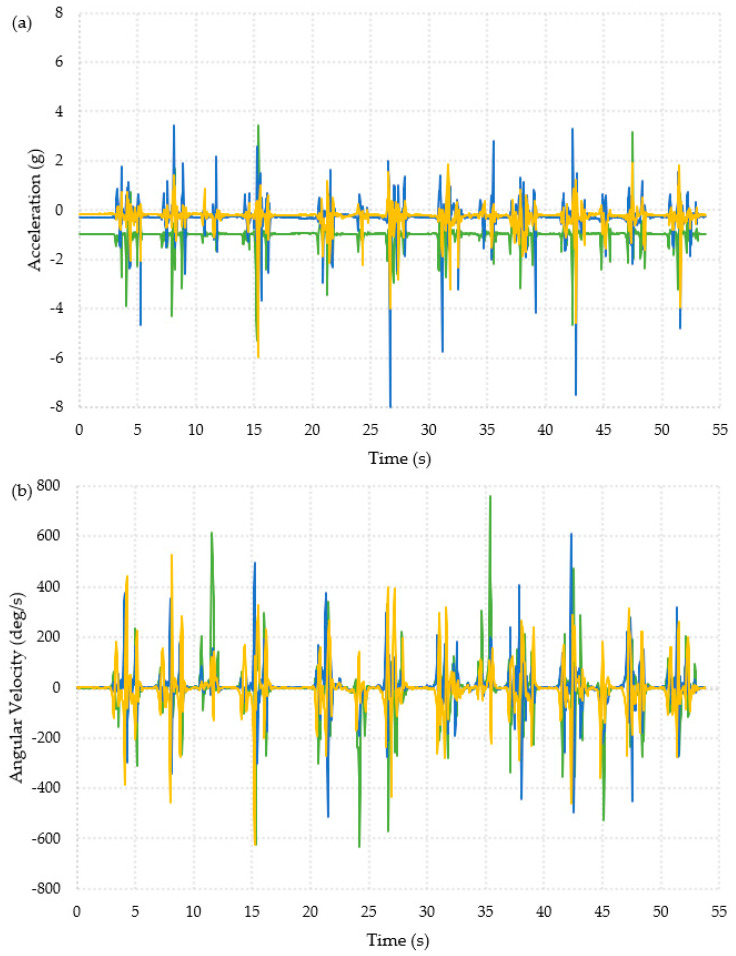
Graphical representation of the data collected from one foot for Participant 3 during the controlled stumble activity. X-axis = 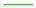, y-axis = 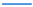, z-axis = 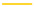. Ten repeated stumbles are shown. (**a**) Acceleration. (**b**) Angular velocity.

**Figure 11:**
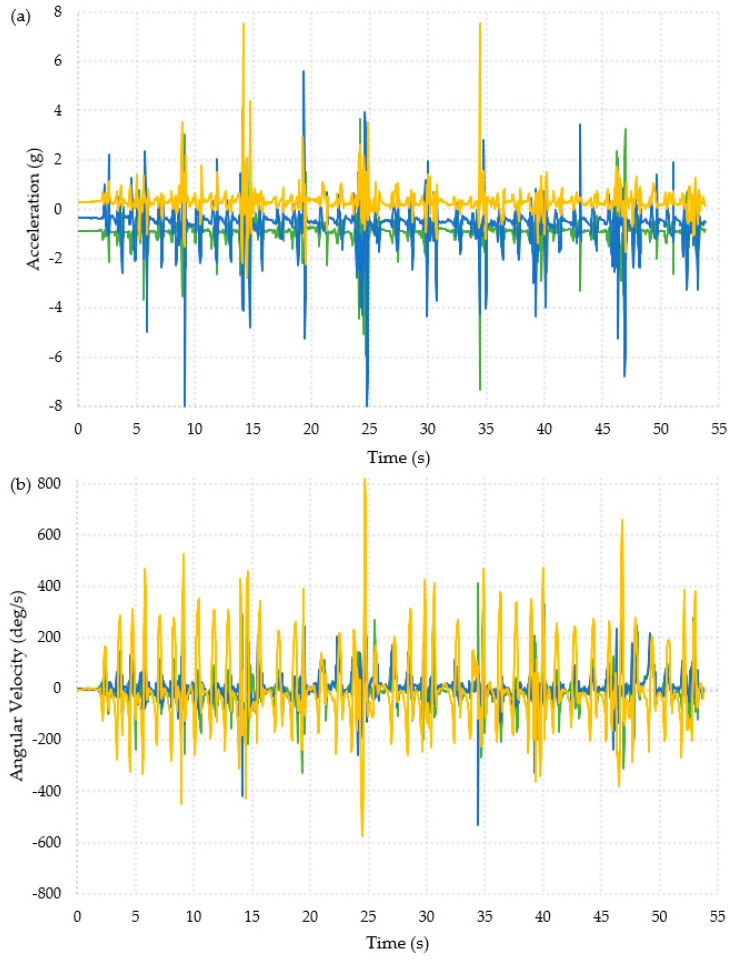
Graphical representation of the data collected from one foot for Participant 11 during the controlled stumble activity. X-axis = 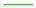, y-axis = 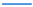, z-axis = 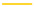. Ten repeated stumbles are shown. (**a**) Acceleration. (**b**) Angular velocity.

The graphs presented below show the data from the controlled stumble activity for participants 3 and 11. This was the only activity where the patterns in the data were not visually observed to be consistent between participants. Data from Participant 3 (Figure 10) and Participant 11 (Figure 11) are presented as they performed the stumble in two different ways.

Figure 10 shows the data for when Participant 3 was stumbling. This participant took a step to stumble and then paused before performing the next stumble. This participant stumbled ten times and turned four times during the activity. The turns can clearly be seen in Figure 9b; when the participant turned, there was either a large increase or decrease in the x-axis angular velocity, seen at 12 s, 24 s, 35 s, and 45 s. This change was much less obvious in the acceleration data.

Figure 11 shows the acceleration and angular velocity data recorded for Participant 11 during the stumbling activity. This participant interpreted the activity differently compared to Participant 3. They continuously walked around the room and completed the ten stumble repeats in that time. Figure 10a shows the acceleration data, and there are large changes in acceleration in the y-axis corresponding to each stumble. The limit of the sensor was set at ±8 g, which the participant exceeded twice. There were also visible changes in the z-axis for each stumble, but these tended to be smaller than the changes in acceleration on the y-axis. The angular velocity data seen in Figure 10b showed large changes in the angular velocity in the z-axis that are larger than those seen during the walking activity, and the acceleration change for the stumble was larger than for falling. This suggested that a threshold value for a fall when the sensor is placed on the ankle would produce false positives if a person stumbled.

**Figure 12:**
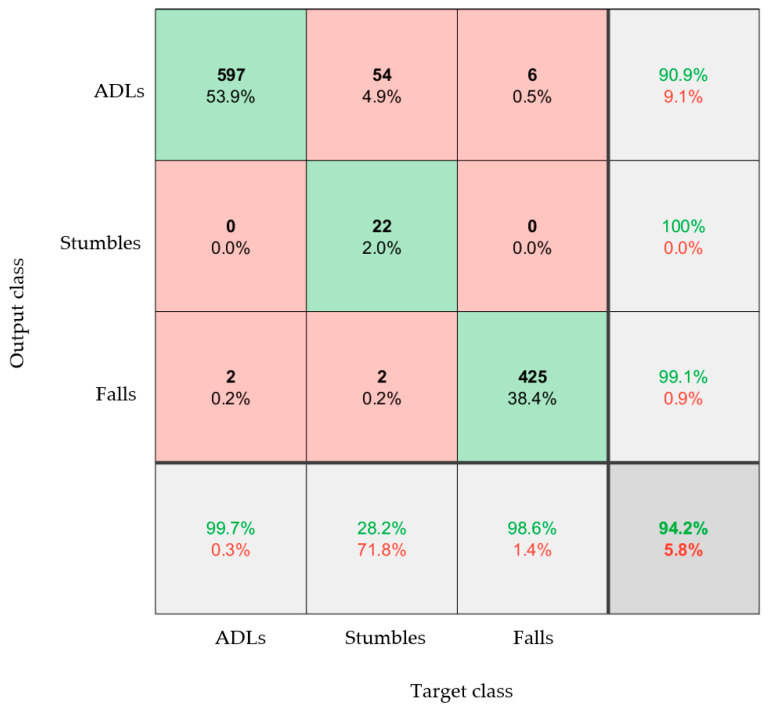
Confusion matrix that classifies between the three types of ADL (walking, sit to stand, timed up and go), the three types of fall, and a near-fall event (stumble). The model was trained and tested using datasets from only the right foot and both acceleration and angular velocity data.

While the types of stumbles conducted by the participants were highly varied, the very high accuracy of the system when comparing fall events and ADLs meant that extracting abnormal events (stumbles) might be possible. This could represent a near-fall in a practical scenario. As the right foot gave the best results (when all the data was used), the model was retrained using three output classes: ADL, fall, and stumble (Figure 12). It should be noted that these datasets were not balanced, as there were significantly fewer stumble events on which to train the model.

Figure 12 clearly showed that stumbles could be identified, distinct from both falls and ADLs, with 94.2% accuracy. This showed that the sock can detect near fall events. Future work will need to repeat these experiments with a greater number of stumbles and near-fall activities to further validate the accuracy of the proposed system.

### 3.4. Feedback on Sock Design

Specific design decisions were made to ensure that the over-sock was both functional and user-friendly. The sock was designed to be comfortable, so cushioning for the E-yarn and hardware were integrated into the knit design (by knitting a spacer structure). Additionally, the opening to the pocket that contained the hardware module was left relatively large for ease of access during the trials, but also because older women are more likely to develop osteoarthritis [21] and rheumatoid arthritis [22] than men, potentially making handling the hardware difficult for them. During the human trial, participants were given the option to provide feedback on the comfort of the sock. The participants found the over-socks comfortable during the trial. Two of the participants initially wore the over-sock inside out and could not feel the hardware. Another participant wore the over-sock upside down and did not notice any discomfort. This implies that it would be best to add a direction to the sock to indicate which way to put it on. One participant mentioned that they liked the over-sock because the electronics were unnoticeable.

## 4. Conclusions

This work presents an over-sock with an embedded IMU that can be used for fall and near-fall detection. By implementing a Bi-LSTM, three different ADLs and three different falls could be differentiated with an accuracy of 85.7%, ADLs and falls could be distinguished with a 99.4% accuracy, and ADLs, falls, and stumbles (near falls) could be classified with a 94.2% accuracy. The work established that the identification of different activities was more successful when only data from one foot was used instead of both. From the results, it is proposed that a monitoring solution of this type be worn on the right foot.

Future work will include repeating the study with additional stumbles, as this dataset was not balanced with the ADLs and falls for this study. Further, with the utility of the device proven, it will be important to test the over-sock on older people, as opposed to the young, healthy volunteers used in this study, as people’s gait patterns are known to change with age. Repeating the study with a male cohort and validating the system for fall and near-fall detection with men will also be part of future work. Ultimately the proposed over-sock could be a powerful tool in helping understand if older adults are at risk of falling.

## Figures and Tables

**Table 1:** Illustration of the bi-directional long term short term memory (BiLSTM) network topology used to classify the data in this work [11].

Sequence input layer
Dropout layer
BiLSTM layer (200 nodes)
Dropout layer
ReLU layer
Fully connected layer
Softmax Layer
Output Layer

**Table 2:** Overall accuracy of the acceleration, angular velocity and both combined on each foot individually and both together. Values have been taken from the confusion matrices.

	Right Foot	Left Foot	Right and Left Foot
Acceleration data	72.1%	73.5%	73.2%
Angular velocity data	85.4%	87.9%	83.9%
Combined acceleration and angular velocity	85.7%	71.6%	81.2%

## Data Availability

The data used for this work can be found on Figshare at https://doi.org/10.6084/m9.figshare.21632078.v1. It also includes all other confusion matrices created for this work.
